# CT Perfusion in the Characterisation of Renal Lesions: An Added Value to Multiphasic CT

**DOI:** 10.1155/2014/135013

**Published:** 2014-08-13

**Authors:** Francesco Giuseppe Mazzei, Maria Antonietta Mazzei, Nevada Cioffi Squitieri, Chiara Pozzessere, Lorenzo Righi, Alfredo Cirigliano, Susanna Guerrini, Domenico D'Elia, Maria Raffaella Ambrosio, Aurora Barone, Maria Teresa del Vecchio, Luca Volterrani

**Affiliations:** ^1^Department of Diagnostic Imaging, Azienda Ospedaliera Universitaria Senese, Viale Bracci 10, 53100 Siena, Italy; ^2^Department of Medical, Surgical and Neuro Sciences, Diagnostic Imaging, University of Siena, Viale Bracci 10, 53100 Siena, Italy; ^3^Department of Molecular and Developmental Medicine, University of Siena, Via Aldo Moro 2, 53100 Siena, Italy; ^4^Department of Medical Biotechnologies, Section of Pathology, University of Siena, Viale Bracci 10, 53100 Siena, Italy; ^5^Department of Medical, Surgical and Neuro Sciences, Section of Pathology, University of Siena, Viale Bracci 10, 53100 Siena, Italy

## Abstract

*Objective.* To prospectively evaluate if computed tomography perfusion (CTp) could be a useful tool in addition to multiphasic CT in renal lesion characterisation. *Materials and Methods.* Fifty-eight patients that were scheduled for surgical resection of a renal mass with a suspicion of renal cell carcinoma (RCC) were enrolled. Forty-one out of 58 patients underwent total or partial nephrectomy after CTp examination, and a pathological analysis was obtained for a total of 49 renal lesions. Perfusion parameters and attenuation values at multiphasic CT for both lesion and normal cortex were analysed. All the results were compared with the histological data obtained following surgery. *Results.* PS and MTT values were significantly lower in malignant lesions than in the normal cortex (*P* < 0.001 and *P* = 0.011, resp.); PS, MTT, and BF values were also statistically different between oncocytomas and malignant lesions. According to ROC analysis, the accuracy, sensitivity, and specificity to predict RCC were 95.92%, 100%, and 66.7%, respectively, for CTp whereas they were 89.80%, 93.35%, and 50%, respectively, for multiphasic CT. *Conclusion.* A significant difference between renal cortex and tumour CTp parameter values may suggest a malignant renal lesion. CTp could represent an added value to multiphasic CT in differentiating renal cells carcinoma from oncocytoma.

## 1. Introduction

Renal cell carcinoma (RCC) represents 3-4% of all malignancies worldwide [1]. It is classified into several types which have different features and clinical behaviours; however histologic type is one of the most important prognostic factors. Clear cell RCC, the most common type, accounting for 65–70%, has a high metastatic potential, whereas papillary RCC (10–15% of RCCs) and chromophobe RCC (5% of RCCs) have a low metastatic potential. The other malignant RCCs account for 5 to 6%. Approximately 20% of renal lesions are benign, and oncocytoma, which accounts for 5% of all renal tumours, is the most common type [2, 3]. RCC's incidence has risen over the last few years because the widespread use of cross-sectional imaging has increased the incidental detection of renal lesions, particularly those of a small size (<4 cm) [4, 5]. Although the great value of imaging for renal lesions detection has increased in recent years, the accuracy rate on preoperative characterisation of their nature remains low [6]; in particular the differential diagnosis of oncocytoma versus RCC represents a diagnostic challenge [7]. Percutaneous biopsy could be a useful tool in dubious cases, but it is an invasive approach [8, 9]. Recently, computed tomography perfusion (CTp), a functional tool which allows a quantitative evaluation of tissue perfusion through consecutive scans acquired during contrast media injection, showed promising results in the oncologic field, even in renal lesion characterisation [10]. It is based on a time-density curve developed by software, but its reliability is still being evaluated. Furthermore the parameters used are not standardised yet, because of the availability of different software platforms and the different version upgrades of the same software, which show different perfusion measurements [11]. The identification of renal lesion type, firstly discriminating between malignant and benign, could represent an important diagnostic goal in order to choose the best management: diagnosis of RCC at an early stage means a less invasive therapeutic approach and a better prognosis, while identifying a benign lesion could avoid any unnecessary surgical intervention. By examining the previous consideration, the aim of our study was to prospectively evaluate if CTp could be a useful tool in addition to multiphasic CT in renal lesion characterisation.

## 2. Materials and Methods

### 2.1. Study Population

Our study had institutional review board approval and a written informed consent was obtained from all patients. Fifty-eight patients scheduled for surgical resection of a renal mass with a suspicion of RCC between April 2012 and December 2013 were considered for the study enrollment. All of these patients underwent renal CTp imaging and staging thoracic and abdominal CT scans. Seventeen patients were excluded as their CTp studies were not evaluable due to significant respiratory artifacts (*n* = 8), or the surgical procedure was performed in another hospital (*n* = 9). Among 58 patients included in this study, 41 (26 males, mean age of 60.76 years, range 39–86 years) underwent total or partial surgical nephrectomy at our hospital within 15.3 days (range: 1–25 days) after CTp examination, and a pathological analysis was obtained for a total of 49 renal lesions.

### 2.2. CT Examination

All patients were examined using a 64-detector row CT scanner (Discovery 750 HD, GE Healthcare, Milwaukee, WI, USA). To reduce respiratory artifacts, a belt over the abdomen was used and patients were instructed to breathe gently during the scan acquisition. An unenhanced CT scan of the upper abdomen covering the kidneys was performed initially to locate the renal lesion. A supervising radiologist (9 years of experience in CTp) identified the tumour and then placed the predefined scan volume (80 mm for shuttle axial technique and 40 mm for cine technique) in the *z*-axis to cover the lesion for the CTp study. Cine technique was used when the lesion was smaller than 20 mm. For the CTp study, 100 mL of Iomeprol (Iomeron 400; Bracco, Milan, Italy) was administered intravenously at a flow rate of 5 mL/s followed by 40 mL of saline solution at the same flow rate. The dynamic cine acquisition consisted of 8 contiguous sections, collimated to 5 mm, with temporal resolution of 1 second by using a cine-mode acquisition without table movement and with the following parameters: 100 Kv, 80 mAs, rotation time 0.5 s, and scan field of view of 50 cm, whereas it consisted of 8 contiguous sections, collimated to 5 mm, with temporal resolution of 2.8 seconds by using a shuttle-mode acquisition with table movement (21 passes) and with the following parameters: 100 Kv, 80 mAs, rotation time 0.4 s, and scan field of view of 50 cm. Total duration time was approximately 60 seconds in order to include both first-pass enhancement and delayed phase. Scanning commenced 6 seconds after the start of the contrast material injection in order to ensure the acquisition of a little nonenhanced baseline data both to allow the software to plot the enhancement change over time and to allow the radiologists to evaluate the lesion's density upon unenhanced CT at the multiphasic CT density evaluation. Immediately after completion of the CTp scans, a conventional diagnostic CT of the abdomen and thorax (CT nephrographic phase, delay of 60 to 80 s, slice thickness 2.5 mm, reconstruction interval 1 mm, collimation 40 mm, beam pitch 0.98, 140 kV, and 200–700 mA with automatic scan exposure) was performed with the intravenous administration of an additional amount of the same nonionic iodinated contrast medium mentioned previously at a rate of 4 mL/sec (up to a total amount of about 150–160 mL of iodinated contrast medium according to the patient body weight), followed by a 20 mL bolus of saline solution administered at the same rate. Finally an excretory phase CT urography was obtained after 5 to 10 minutes of the contrast media injection for 32 out of the 41 patients. All the CT scans were performed by using the adaptive statistical iterative reconstruction (ASIR, 30%) system in order to reduce the dose exposure to the patient.

### 2.3. Image Analysis

Image analysis was performed in consensus by a radiologist and a resident fellow in radiology (with 9 and 2 years of experience in CTp, resp.). All CTp studies were analysed by using a commercial perfusion software (Body Tumor CT Perfusion Software version 3; GE Healthcare). For the CTp analysis, a processing threshold (CT value range) between 0 and 120 Hounsfield units (HU) was utilised to optimise visualisation of the soft tissue. On transverse CT images, the slice showing the maximal transverse tumour diameter was chosen for further analysis. The arterial input was determined by placement of a circular region of interest (ROI) in the abdominal aorta, to measure the arterial input function. An arterial time-density curve (TDC) for the entire acquisition time of each study was generated automatically. In the same selected image, ROIs of the renal tumour and normal renal cortex were drawn manually (maximum 1 cm^2^), lying within the structure of interest in each slice and excluding necrosis, calcifications, or cystic or any hemorrhagic areas. Mean values for four CTp parameters (permeability surface, PS; mean transit time, MTT; blood volume, BV; and blood flow, BF) were obtained and recorded for each patient. For the multiphasic evaluation, both the baseline noncontrast phase and the corticomedullary phase (CM) were evaluated on the CTp images: in particular CM phase imaging occurred 35 seconds after the threshold level of 150 HU was reached in the ROI placed in the aorta. The nephrographic phase (NG) imaging occurred 60 to 80 seconds after the threshold level of 150 HU was reached and the excretory phase imaging occurred 5 to 10 minutes after the threshold level of 150 HU was reached. On the basis of the normal appearance of renal parenchyma, images from these protocols were classified as unenhanced if there was no contrast material administration, corticomedullary if the renal cortex but not the medulla enhanced in a ribbon-like pattern, nephrographic if the cortex and medulla enhanced uniformly, or excretory if the concentrated contrast material was excreted in the renal pelvis and ureters after the prior phases. Average tumour attenuation measurements were determined in each phase; average cortical and aorta attenuation measurements were taken in the same image in which the tumour attenuation was determined.* Absolute enhancement* was defined as the difference in mean HU between the noncontrast phase and any given contrast phase (CM, NG, or excretory);* percentage enhancement* was calculated as the mean HU in the tumour divided by the mean HU of the tumour in the noncontrast phase.* Relative enhancement to renal cortex* (or cortical-tumour relative enhancement) was defined as the difference between mean tumour enhancement and renal cortical enhancement during a given phase, whereas the cortical-tumour ratio was defined as mean tumour enhancement divided by renal cortical enhancement during any given phase;* relative enhancement to aorta* (or aorta-tumour relative enhancement) was also calculated as the difference between mean tumour enhancement and aorta enhancement during a given phase, whereas the aorta-tumour ratio was defined as mean tumour enhancement divided by aorta enhancement during any given phase. For each patient, aorta, cortical, and lesion ROIs were fixed in the same location and in the same axial slice level to subsequently enable an identical placement at the same lesion axial slice level for the multiphasic analysis by saving the ROIs within the software platform. The ROIs were reviewed by each reader for appropriate placement. The maximal diameter of each lesion was measured on axial images, and this measurement was reviewed by each reader; renal lesions were defined “hypervascular” if enhancement in the nephrographic phase was greater than or equal to that of renal cortex (in HU density) [12].

### 2.4. Histopathology

The surgical specimen consisted of radical nephrectomy in 31 out of 41 (75%) patients and partial nephrectomy in 10 out of 41 (25%) patients. In addition to routine samples for pathologic diagnosis, additional tissue blocks from each tumour as well as from normal renal tissue were acquired for additional histological examination and immunohistochemical staining by the pathologist. The pathologist took care to ensure sampling at a tumour level corresponding to the level at which CTp was performed. In particular on CT images the distance between the CT slice showing the maximal transverse tumour diameter and the lower or upper pole of the kidney or, in case of partial nephrectomy, the lower or upper margin of the tumour was measured. One pathologist and one radiologist (who supervised the CTp study) jointly performed the processing of all surgical specimens and reported the coordinates of the slice analysed on CTp images. All tissue specimens were fixed in a 10% buffered formalin and embedded in paraffin. The surgical specimens were sliced in the transverse plane at the level of the maximal tumour diameter, according to the distance measured on CT images. The macroscopic appearance of the transversally sliced surgical specimen was compared with the appearance of the corresponding tumour plane on transverse CT images to ensure that they were similar. From each block, 4-micron-thick sections were cut. The sections were stained with hematoxylin and eosin. All tumours were staged based on the last TNM classification system (TNM7) and the 4-tiered Fuhrman grading system was used to grade the tumours (MTdV). Quantification of microvessel density (MVD) was performed after immunostaining with a CD34 monoclonal antibody (clone QBEnd/10, ready to use) by light microscopy using the counting method introduced by Weidner et al. [13, 14] and stained with CD34 for quantification of MVD. The staining was performed on a Bond Max automated immunostainer (Leica Microsystem, Bannockburn, IL, USA) by a monoclonal antibody (predilute AP 125; ProgenBiotechnik GmbH, Maabstrasse, Heidelberg, Germany) with controls in parallel. No epitope retrieval was used. Ultravision Detection System using antipolyvalent HRP (LabVision, Fremont, CA, USA) and diaminobenzidine (DAB, Dako, Milan, Italy) as chromogen was used. Briefly, the whole slide was viewed at ×100 magnification and the area containing the maximum number of microvessels (the “hotspot” area) was identified. The precise topography of the angiogenic hotspots in carcinomas was assessed by measuring their distance from the tumour edge; hotspots within 0.5 mm were considered marginal. Then, under x400 magnification (where one field is equivalent to 0.19 mm^2^) individual microvessels were counted. The number of vessels in six areas was counted and averaged as MVD. Both isolated immunoreactive endothelial cells and luminal microvascular structures were considered countable vessels. Distinct endothelial cell staining of the renal vasculature served as a positive control. Occasional immunoreactive macrophages and plasma cells were excluded, based on their morphological appearance. Assessment of MVD was completed without knowledge of any clinicopathological data and blinded to the results from CT perfusion imaging. Two pathologists (MRA and BJR) counted MVD, respectively, and mean values were calculated and recorded for each patient. Disagreements on what constituted a microvessel were resolved by consensus.

### 2.5. Statistical Analysis

Shapiro Wilk test was used to test the normality of variables. CTp parameters in tumour tissue and adjacent normal parenchyma were compared using Wilcoxon signed-rank test; differences in CTp parameters and multiphasic CT measurements between benign and malign tumours and between benign and malign hypervascular tumours were assessed by using the Wilcoxon-Mann-Whitney test; Kruskal-Wallis test was used to compare CTp parameters between the three malignant histologic subtypes; a *P* value less than 0.05 indicated a statistically significant difference. Spearman test was used to evaluate the correlation between MVD and selected CTp and multiphasic CT measurements. For this analysis, the Bonferroni correction was used and the significance level was set to 0.0071. Diagnostic accuracy of variables was measured by using receiver operating characteristic (ROC) analysis. ROC curves were analysed to determine the cutoffs that maximise the number of correctly classified lesions. All analyses were carried out using STATA statistical software V.12.1 (StataCorp, Texas).

## 3. Results

All patients underwent CTp imaging without any adverse effects. Three out of 41 patients had multiple lesions; more specifically, of the 3 patients who had multiple lesions, 1 had six lesions, 1 had three lesions, and 1 had two lesions, for a total of 49 renal lesions. Of the 49 renal lesions included in this study, 27 (55%) were clear cell RCCs, 10 (21%) were chromophobe RCCs, 6 (12%) were papillary RCCs, and 6 (12%) were oncocytomas. Mean lesion diameters were 51.25 mm for clear cell RCCs, 44.2 mm for chromophobe RCCs, 26.6 mm for papillary RCCs, and 48.5 mm for oncocytomas. The pathologic tumour stage and baseline characteristics for each of the groups are presented in Table 1. Forty out of 49 lesions (82%) had been imaged also with an excretory CT scan after the CTp study (32 out of 41 patients). 25 out of 49 lesions (51%) were hypervascular at CT examination (17 clear cell RCCs, 2 chromophobe RCCs, 2 papillary RCCs, and 4 oncocytomas), 14 out of 49 lesions (29%) were hypervascular with a necrotic core (10 clear cell RCCs, 2 chromophobe RCCs, and 2 oncocytomas), and 10 (20%) were hypovascular (6 chromophobe RCCs and 4 papillary RCCs).

### 3.1. CTp Measurements

**Figure 1:**
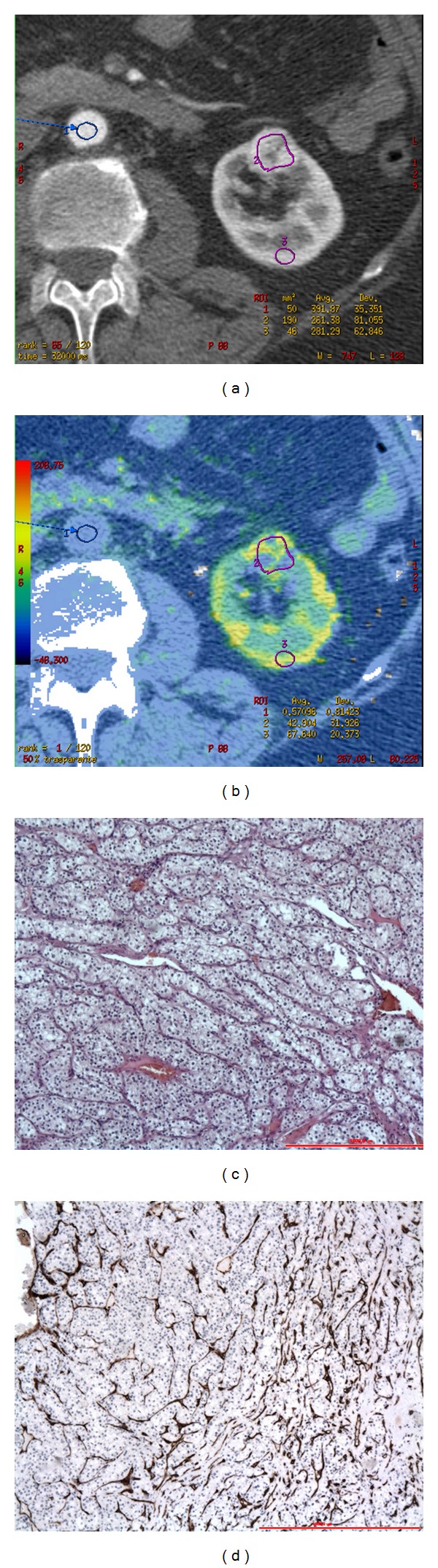
CTp of clear cell RCC: ROI 1, ROI 2, and ROI 3 were drawn in the aorta, tumour, and healthy ipsilateral renal cortex, respectively (a-b). The PS values of the tumour and normal cortex were 42.90 mL/100 g/min and 67.64 mL/100 g/min, respectively, whereas MTT values were 13.73 sec and 3.59 sec, respectively (b). Lesion size was 20 mm. Histopathology: morphology, haematoxylin, and eosin (c) and CD34 stain (d); original magnification: 50x.

**Figure 2:**
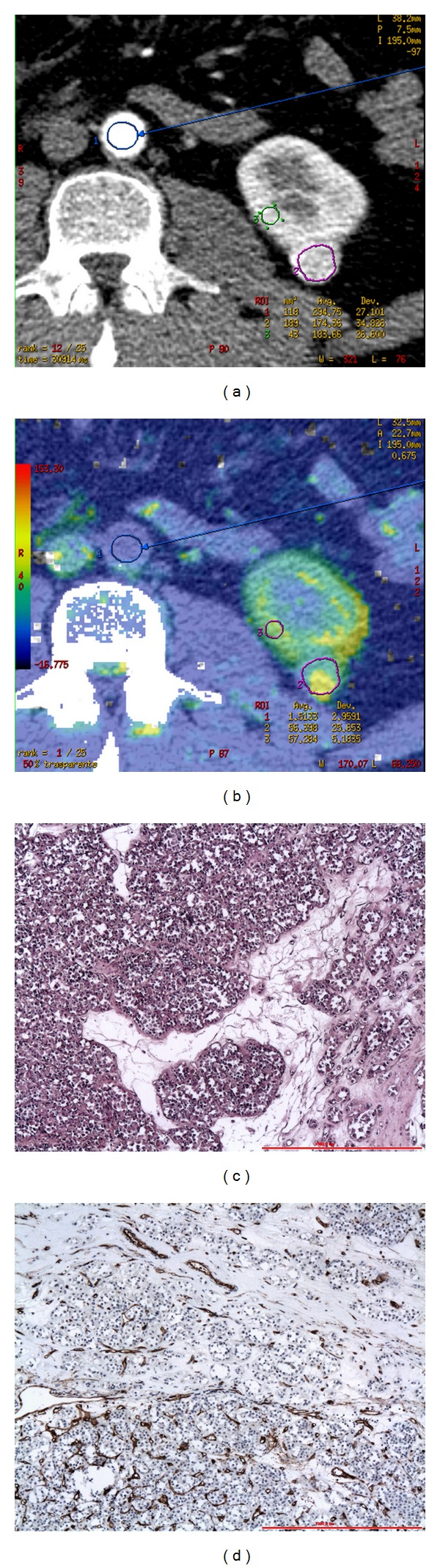
CTp of oncocytoma: ROI 1, ROI 2, and ROI 3 were drawn in the aorta, tumour, and healthy ipsilateral renal cortex, respectively (a-b). The PS values of the tumour and normal cortex were 56.39 mL/100 g/min and 57.28 mL/100 g/min, respectively, whereas MTT values were 10.48 sec and 9.65 sec, respectively (b). Lesion size was 20 mm. Histopathology: morphology, haematoxylin, and eosin (c) and CD34 stain (d); original magnification: 50x.

**Figure 3:**
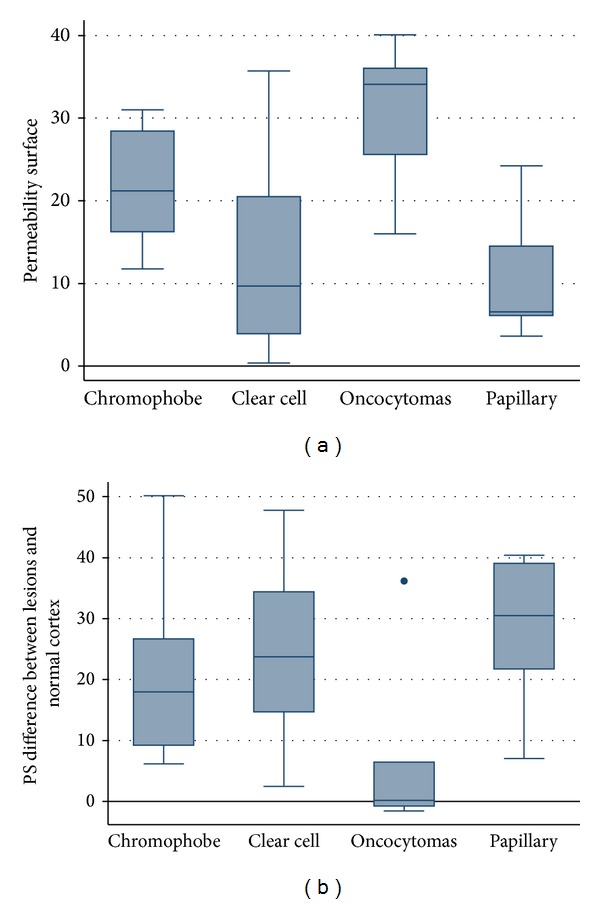
Box whiskers graphics: permeability surface values in different histological type of renal lesions (a); difference of permeability surface values between lesion and normal cortex in different histological type of renal lesions (b). The boxes display the interquartile range (the 25th and 75th percentile) and the median of CTp measurements for the four histologic subtypes. The whiskers display the upper and lower values within 1.5 times the interquartile range beyond the 25th and 75th percentile. Any outliers beyond those limits get their own markers (dot mark).

Mean perfusion CT parameter values (PS, MTT, BV, and BF) for the normal renal cortex and renal tumours (oncocytomas and malignant lesions) are summarised in Table 2. There were significant differences in PS (*P* < 0.001) and MTT (*P* = 0.011) between tumour and normal renal cortex in malignant lesions (clear cell carcinoma, chromophobe carcinoma, and papillary carcinoma) whereas there were no differences in any CTp parameters between lesion and normal renal cortex in oncocytomas (Figures 1 and 2). There were also significant differences in PS, MTT, and BF parameters between oncocytomas and all malignant renal lesions and in PS and MTT between oncocytomas and malignant hypervascular lesions, with or without necrosis (Table 3). Significant differences were also found in PS (*P* = 0.0137), BV (*P* = 0.0106), BF (*P* = 0.0258), and MTT (*P* = 0.0084) among the three different pathologic types of malignant lesions. The CTp parameter with the highest capacity for discriminating benign from malignant lesions, evaluated through ROC analysis, was PS (maximum accuracy 93.88%). The difference between the normal cortex and tumoural PS values yielded an even more accurate result; with 2.5 mL/100 g/min as a cutoff we achieved a sensitivity, specificity, and accuracy of 100%, 66.67%, and 95.92%, respectively, to predict RCCs. The results of the ROC analysis regarding the comparison between oncocytomas and all malignant lesions PS and between oncocytomas and malignant hypervascular lesions PS are illustrated in Table 4. The box and whisker plots regarding the absolute PS and the difference between normal cortex and tumoural PS values of different histologic types of tumour are presented in Figure 3.

### 3.2. Attenuation Measurements

**Figure 4:**
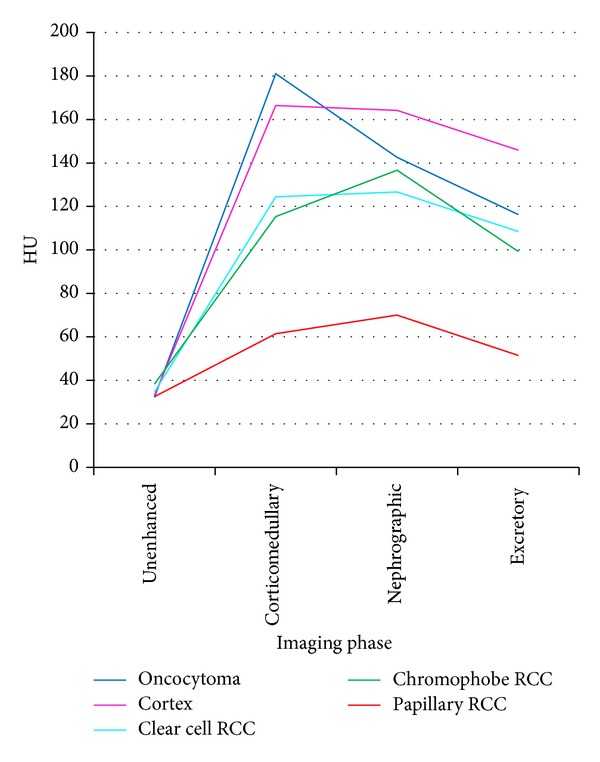
Patterns of enhancement on multiphasic imaging of 49 renal masses by tumour histology.

Graphs and data depicting enhancement patterns are demonstrated in Figure 4. Significant differences between oncocytomas and malignant renal lesions were noted in absolute (*P* = 0.0087) and percentage (*P* = 0.0061) enhancement in the CM phase. Significant differences in absolute (*P* = 0.0265) and percentage (*P* = 0.0158) enhancement in CM phase were also noted, among hypervascular lesions and in particular among oncocytomas and malignant lesions. The multiphasic CT measure with the greatest accuracy for discriminating benign from malignant lesions was an absolute enhancement in CM phase. Using ROC analysis, the optimal cutoff value in this study was ≤160 HU enabling a sensitivity, specificity, and accuracy rate of 95.3%, 50%, and 89.8%, respectively, to predict RCCs.

### 3.3. MVD Analysis

The CTp parameters BF and BV measured in the tumour cross-section ROI showed a positive correlation with tumour cross-section MVD in malignant lesions (*P* = 0.006 and *P* = 0.0026, resp.) but not in oncocytomas. No significant correlations were found between other CTp or multiphasic parameters (absolute enhancement in CM, NG, or excretory phase) and the tumour cross-section MVD.

## 4. Discussion

RCC represents a different spectrum of disease with different features and clinical behaviours, largely depending on the histologic type. The most aggressive type is clear cell RCC whereas papillary RCC and chromophobe RCC can both be considered indolent, because of their low metastatic potential. However differential diagnosis of renal masses also includes benign lesions (about 20%) and oncocytoma is the most common type [2, 3]. Over the last few years, the introduction of nephron-sparing nephrectomy has changed the approach to early stage renal cancer, allowing reduced complication rates with survival rates similar to total nephrectomy. In this sense, renal lesions characterisation should be mandatory in order to recognise a small RCC which could be treated through a partial nephrectomy rather than a wait and see approach. Moreover, differentiating benign lesions is important in order to avoid unnecessary surgery. Despite the widespread use of multimodality imaging, the characterisation of renal lesion still remains poor in some cases, particularly those of small size (<40 mm) [4–6]. In fact, while papillary RCC typically shows low contrast-enhancement at CT, oncocytoma and clear cell RCCs can have similar features and postcontrastographic behaviour, making a differential diagnosis difficult when it should be mandatory [7, 15, 16]. Several studies, using multiphase CT technique, identified differing degrees of enhancement in different postcontrast phases as the most reliable parameter to distinguish clear cell RCCs from other subtypes, including oncocytomas [16–18]. According to these studies, we also report differing degrees of contrast-enhancement between clear cell RCCs and other subtypes, including oncocytomas; however in contrast with them, in our study, as in the study of Gakis et al., oncocytoma presented a wash-in similar to renal cortex and clear cell RCCs had a lower enhancement than oncocytomas during any given phase, whereas they described a higher contrast-enhancement as a distinctive feature for discriminating clear cell RCCs in relation to oncocytomas [18–20]. Moreover, in our study, as previously reported by others, we identified that both clear cell RCCs and oncocytomas could show two different morphological features, being homogeneously hypervascular (17 and 4 cases, resp.) and hypervascular with a necrotic core (10 and 2 cases, resp.); in a few cases, even chromophobe RCCs and papillary RCCs had the same CT appearances [7, 16]. For this reason, we tested both multiphasic CT and CTp in discriminating malignant lesions in this subgroup. Furthermore still in this subgroup (hypervascular lesions), absolute percentage enhancement in CM phase was significantly higher in oncocytomas than in malignant lesions (*P* = 0.02 and *P* = 0.01, resp.), and ROC analysis showed an accuracy rate of 94% using <160HU in absolute enhancement as a cutoff for identification of malignant lesions. No significant differences in lesion-cortex ratio or in lesion-aorta ratio were identified in any given phase. To the best of our knowledge, this is the first study which has compared the same series of lesions by using both multiphasic CT and CTp. CTp allows the quantitative evaluation of a tissue perfusion whilst also optimising the acquisition protocol and has shown promising results in the oncologic field, even in RCC characterisation [10, 21, 22]. Like the recent studies, we found significant differences in PS and MTT values between malignant lesions (clear cell RCCs, papillary RCCs, and chromophobe RCCs) and the normal renal cortex (*P* < 0.001 and *P* = 0.029, resp.); however in our study BF and BV values were not significant; moreover, none of the CTp parameters evaluated in oncocytomas demonstrated significant differences from those of a normal renal cortex [10, 23, 24]. We also report significant lower values, in PS, MTT, and BF values (*P* < 0.001, *P* = 0.01, and *P* = 0.02, resp.) in malignant lesions in comparison with oncocytomas, and these results were confirmed for PS and MTT values considering only the hypervascular subgroups (those with a necrotic core versus those homogeneously hypervascular). These results could be explained by alterations of microvessel architecture in RCC, whereas oncocytomas, instead, appear to exhibit normal architecture, very close to the normal renal cortex. In fact, by evaluating a possible correlation of perfusion parameters with histological findings, particularly with microvessel density (MVD), a prognostic marker of RCC, we found a significant correlation between BF and BV and MVD (*P* < 0.01), suggesting that these CTp parameters may reflect blood vessels and then neoangiogenesis of RCCs, as also suggested by previous studies [10, 24]. At ROC curve analysis, the difference between normal cortex and tumoural PS values yielded the best result with a cutoff greater than 2.5 mL/100 g/min with a sensitivity, specificity, and accuracy of 100%, 66.67%, and 95.92%, respectively, to predict RCCs. No increased accuracy rate was obtained by considering both multiphase CT and CTp analysis. Previous studies tried to describe and differentiate the renal lesions according to the morphological criteria alone, which is often possible in large lesions, but which can be difficult for small lesions, or according to different multiphasic enhancement patterns upon CT examination, or using CTp parameters alone [10, 16–20]. One of the unique elements of our study is that it is the first which considers two different CT techniques in the evaluation of the same series of renal tumours; however it has some limitations. Firstly, the number of renal lesions is low (49 lesions), and the subgroup of benign lesions included is relatively small (6 lesions, 12%). This could explain the low sensitivity and specificity rates obtained by ROC analysis in both multiphasic CT and CTp analyses, respectively. Increasing the case history could strengthen the results. Furthermore some common renal lesions, like angiomyolipoma with minimal fat, are not represented in our case population. Secondly, only 49% of the lesions are small renal lesions (<40 mm), which are those with uncertain management: some authors, in fact, claimed that a followup with an active surveillance instead of surgical excision should be considered in these cases, in particular if old age, decreased life expectancy, or extensive comorbidities are associated [25]. Furthermore papillary RCCs are significantly smaller than other tumours and this could influence the vascularisation patterns, even if several studies reported similar enhancement patterns for both small and large papillary RCCs [26]. Finally the CTp showed some limitations. Patients compliance is needed; in our experience 8 patients were excluded because of respiratory artifacts; moreover, it is not standardised yet, depending on several factors including the hardware platform and software algorithm [11, 27].

## 5. Conclusion

This study, even with the limitations just considered, showed the feasibility of CTp in discriminating between renal cell carcinoma and oncocytoma, which can be an aid in management of renal lesions. In particular we would like to state not only a standard cutoff for CTp analysis, but also a significant difference between renal cortex and tumour CTp parameter values that may suggest a malignant lesion. In an era with increased interest in active surveillance, future studies will determine whether adding CTp measurements could further refine our predictive ability in the characterisation of renal masses.

## Figures and Tables

**Table 1:** Characteristics of patients, renal lesions, and CT examination.

Characteristics	All lesions (% or range)	Clear cell RCC (% or range)	Chromophobe RCC (% or range)	Papillary RCC (% or range)	Oncocytoma (% or range)
*N* patients	41	26∗	5	5∗	6
Sex					
Male	26 (63)	16 (62)	3 (60)	5 (100)	3 (50)
Female	15 (37)	10 (38)	2 (40)	0	3 (50)
Mean age	60.76 (39–86)	65.29 (39–86)	53.4 (39–60)	58.16 (42–63)	64.5 (44–80)
*N* lesions	49	27 (55)	10 (21)	6 (12)	6 (12)
Side					
Right	25 (51)	14 (52)	6 (60)	3 (50)	2 (33)
Left	24 (49)	13 (48)	4 (40)	3 (50)	4 (67)
Location					
UP	10 (20)	6 (22)	2 (20)	2 (33)	0
UP-MR	5 (10)	3 (11)	1 (10)	0	1 (17)
MR	15 (31)	7 (26)	4 (40)	3 (50)	1 (17)
LP-MR	7 (14)	6 (22)	0	0	1 (17)
LP	12 (25)	5 (19)	3 (30)	1 (17)	3 (49)
Mean lesion diameter (mm)	45.62 (10–128)	51.25 (20–128)	44.2 (10–110)	26.66 (17–40)	48.5 (20–116)
Pathologic tumour stage					
T1a	20 (46)	9 (33)	5 (50)	6 (100)	—
T1b	8 (19)	6 (22)	2 (20)	0	—
T2a	2 (5)	0	2 (20)	0	—
T2b	2 (5)	1 (4)	1 (10)	0	—
T3a	8 (19)	8 (30)	0	0	—
T3b	3 (6)	3 (11)	0	0	—
T3c	0	0	0	0	—
T4	0	0	0	0	—
Fuhrman grade					
1	4 (12)	3 (11)	—	1 (17)	—
2	21 (64)	17 (63)	—	4 (66)	—
3	6 (18)	5 (19)	—	1 (17)	—
4	2 (6)	2 (7)	—	0	—
MVD					
Lesion	439.20 (105–1230)	488.66 (238–1230)	412 (105–769)	256 (108–482)	444.3 (288–492)
Parenchyma	266.97 (71–537)	222.29 (80–238)	506.3 (80–537)	240.16 (71–458)	262.6 (162–322)
Perfusion1 CT study type					
Cine	13 (26)	8 (30)	1 (10)	2 (34)	2 (34)
Shuttle	36 (74)	19 (70)	9 (90)	4 (66)	4 (66)
Multiphase CT study type					
B-CM-NG-E	40 (82)	22 (81)	8 (80)	4 (66)	6 (100)
B-CM-NG	9 (18)	5 (19)	2 (20)	2 (34)	0
CT characteristics					
Hypervascular	25 (51)	17 (63)	2 (20)	2 (34)	4 (66)
H with necrosis	14 (29)	10 (37)	2 (20)	0	2 (34)
Hypovascular	10 (20)	0	6 (60)	4 (40)	0

There were 49 lesions in 41 patients; ∗one patient had two different histologic types of lesions: one clear cell RCC and one papillary RCC. Oncocytomas were not staged because the staging criteria only applied to RCCs. RCC: renal cell carcinoma; *N*: number; UP: upper pole; MR: mesorenal region; LP: lower pole; MVD: microvascular density; B: baseline phase; CM: corticomedullary phase; NG: nephrographic phase; E: excretory phase; H: hypervascular.

**Table 2:** Comparison among the CTp parameters: lesions versus normal cortex.

CTp parameters	MLs	NRC	*P* value	Oncocytomas	NRC	*P* value
PS	14.21	38.47	<0.001	35.98	37.74	0.6002
MTT	6.73	4.19	0.011	2.57	2.72	0.7532
BV	15.57	17.08	0.29	18.90	15.02	0.7532
BF	302.87	351.72	0.17	477.02	434.21	0.3454

MLs: malignant lesions; NRC: normal renal cortex; PS: permeability surface; MTT: mean transit time; BV: blood volume; BF: blood flow; a *P* value less than 0.05 indicated a statistically significant difference.

**Table 3:** Comparison among the CTp parameters: malignant lesions versus oncocytomas.

CTp parameters	Oncocytomas	All MLs	*P* value	Oncocytomas	MHLs	*P* value
PS	35.98	14.21	<0.001	35.98	13.91	0.009
MTT	2.57	6.73	0.0109	2.57	6.08	0.0279
BV	18.90	15.57	0.2111	18.90	17.10	0.4596
BF	477.02	302.87	0.0240	477.02	344.55	0.0868

MLs: malignant lesions; MHLs: malignant hypervascular lesions; PS: permeability surface; MTT: mean transit time; BV: blood volume; BF: blood flow; a *P* value less than 0.05 indicated a statistically significant difference.

**Table:** (a) ROC analysis using difference between normal cortex and tumoural PS values

	Oncocytomas versus MHLs	Oncocytomas versus all MLs
Area under ROC curve	0.85 (C.I. 0.59–1)	0.85 (C.I. 0.60–1)
Threshold value(s)	NC PS-tumour PS >2.5	NC PS-tumour PS >2.5
Sensitivity	100%	100%
Specificity	66.67%	66.67%
Accuracy	94.87%	95.92%

**Table:** (b) ROC analysis using absolute enhancement in corticomedullary phase

	Oncocytomas versus MHLs	Oncocytomas versus all MLs
Area under ROC curve	0.79 (C.I. 0.61–0.96)	0.83 (C.I. 0.69–0.97)
Threshold value(s)	<160	<160
Sensitivity	93.94%	95.35%
Specificity	50%	50%
Accuracy	87.18%	89.80%

Results of ROC curve analysis in predicting RCCs in relation to oncocytomas using difference in PS values and absolute enhancement in corticomedullary phase. PS: permeability surface; MHLs: malignant hypervascular lesions; MLs: malignant lesions; NC PS: normal cortex permeability surface.
